# Therapeutic potential of CPI-613 for targeting tumorous mitochondrial energy metabolism and inhibiting autophagy in clear cell sarcoma

**DOI:** 10.1371/journal.pone.0198940

**Published:** 2018-06-07

**Authors:** Yuki Egawa, Chiemi Saigo, Yusuke Kito, Toshiaki Moriki, Tamotsu Takeuchi

**Affiliations:** 1 Department of Pathology and Translational Research, Gifu University Graduate School of Medicine, Gifu, Japan; 2 Division of Pathology, Shizuoka City Shizuoka Hospital, Shizuoka, Japan; University of South Alabama Mitchell Cancer Institute, UNITED STATES

## Abstract

Clear cell sarcoma (CCS) is an aggressive type of soft tissue tumor that is associated with high rates of metastasis. In the present study, we found that CPI-613, which targets tumorous mitochondrial energy metabolism, induced autophagosome formation followed by lysosome fusion in HS-MM CCS cells *in vitro*. Interestingly, CPI-613 along with chloroquine, which inhibits the fusion of autophagosomes with lysosomes, significantly induced necrosis of HS-MM CCS cell growth *in vitro*. Subsequently, we established a murine orthotropic metastatic model of CCS and evaluated the putative suppressive effect of a combination of CPI-613 and chloroquine on CCS progression. Injection of HS-MM into the aponeuroses of the thigh, the most frequently affected site in CCS, resulted in massive metastasis in SCID-beige mice. By contrast, intraperitoneal administration of CPI-613 (25 mg/kg) and chloroquine (50 mg/kg), two days a week for two weeks, significantly decreased tumor growth at the injection site and abolished metastasis. The present results imply the inhibitory effects of a combination of CPI-613 and chloroquine on the progression of CCS.

## Introduction

Clear cell sarcoma (CCS) affects the deep soft tissues of young adults and is known to have high rates of metastasis [1, 2]. Lymphatic metastasis is rare in other malignant soft tissue tumors but is commonly detected in CCS [3, 4]. Radical surgical resection is the first line of treatment of CCS. However, the rate of local recurrence can reach as high as 84% and the rate of late metastases can be as high as 63%, and these are associated with the 5–20-year survival rate of 67–10% [5]. Considering that CCS is relatively resistant to conventional soft tissue sarcoma chemotherapy regimens, there is an urgent need to develop therapies that also control metastasis.

CPI-613 is a first-in-class agent that is an analog of α-lipoic acid coenzyme and targets tumor mitochondrial energy metabolism [6]. CPI-613 can also induce a burst of mitochondrial reactive oxygen species in cancer cells [7]. In line with this concept, CPI-613 specifically kills tumor cells without exhibiting toxicity to primary normal cell counterparts [7]. Serial clinical trials on CPI-613 have been completed for hematological malignancies and pancreatic cancer, and the drug was well tolerated in patients [8, 9].

In this report, we describe the putative suppressive efficacy of CPI-613 on the local and metastatic growth of CCS using a novel murine model with high rates of metastasis.

## Materials and methods

### HS-MM clear cell sarcoma cell line

A CCS cell line, HS-MM, was previously established and characterized in our laboratory [10, 11]. HS-MM cells harbor a canonical genetic background with t(12;22)(q13;q12) of clear cell sarcoma, which results in an *EWS RNA binding protein 1 (EWSR1)-activating transcription factor 1 (ATF1)* fusion gene [12]. Cells were cultured with Dulbecco’s modified Eagle’s medium (DMEM; Gibco Life Technologies, Grand Island, NY, USA) containing 10% heat-inactivated fetal bovine serum.

### CPI-613 and chloroquine, autolysosome detection, and double staining with annexin V and propidium iodide

CPI-613 and chloroquine were purchased from AdooQ BioScience (Irvine, CA, USA) and Nacalai Tesque (Tokyo, Japan), respectively. Necrostatin-1 was purchased from Abcam (Cambridge, UK).

To detect autolysosomes, we employed the DALGreen agent (Dojindo Co., Kumamoto, Japan) according the manufacturer’s protocol. Briefly, DALGreen, which is a small hydrophobic molecule, passes the cell surface membrane of live cells and is incorporated in the autophagosome. After a lysosome fuses with the autophagosome, the incorporated DALGreen begins to fluoresce as the acidity increases [13], and this was visualized under a confocal fluorescence microscope (Leica TCS SP8; Leica Corporation, Germany) and analyzed with a Guava EasyCyte cell analyzer (Hayward, CA, USA).

Cells were also stained with a fluorescein isothiocyanate (FITC)-conjugated Annexin V and propidium iodide (PI) (PromoCell GmbH, Heidelberg, Germany) followed by analysis with a confocal fluorescence microscope and cell analyzer.

### Xenoplantation and CPI-613 treatment

The experimental protocol was approved by the Animal Care Committee of Gifu Graduate School of Gifu, Japan (approval No. 27–80). SCID-beige (CB17.Cg-PrkdcscidLystbg-J/CrlCrlj) mice were purchased from Charles River Laboratories Japan (Sizuoka, Japan) and were housed in the Animal facility of the Gifu Graduate School of Gifu, Gifu, Japan. Mice were monitored for signs of distress and were euthanized humanely according to the guidelines for housing mice in animal facility (100% CO_2_ inhalation followed by cervical dislocation). We did not observe any signs of distress, including weight loss, appetite loss, or tumor ulceration, in any groups of mice in this study. No animal died during the experiment except when sacrificed at the experimental end point.

SCID-beige (CB17.Cg-PrkdcscidLystbg-J/CrlCrlj) mice were purchased from Charles River Laboratories Japan (Sizuoka, Japan).

Mice were injected with 2.5 × 10^7^ HS-MM cells into the aponeuroses of the thighs. Tumor volume was measured by calipers using following equation: tumor volumes (mm^3^) = 4/3 π×[a/2]×[b/2]^2^, where ‘a’ and ‘b’ correspond to the longest and shortest diameter.

Mice were intraperitoneally injected with CPI-613 (25 mg/kg) and chloroquine (50 mg/kg), two days a week for two weeks, when tumor volumes reached approximately 2mm^3^. One week after the last CPI-613 and chloroquine injection, mice were sacrificed to examine their metastatic status. Euthanasia was performed under anesthesia and every effort was made to minimize suffering.

### Reverse transcription polymerase chain reaction (RT-PCR)

cDNA synthesis from total RNA and subsequent PCR were performed using the Reverse Transcription Polymerase Chain Reaction Kit (Takara, Ohtsu, Japan) according to the manufacturer’s instructions and as previously described [14]. The sense and antisense primers used to amplify the fusion gene were 5'-CCCACTAGTTACCCACCCCA-3' (*EWSR1* exon *7*) and 5'-AAAACTCCACTAGGAAATCCATTT-3' (*ATF* exon *4*), respectively. The sense and antisense primers used to amplify glycerol-3-phosphate dehydrogenase (*G3PDH*) were 5'-TCCACCACCCTGTTGCTGTA-3' and 5'-ACCACAGTCCATGCCATCAC-3', respectively. PCR products were subjected to 1% agarose gel electrophoresis.

### Histological analysis, antibodies, and immunohistochemical staining

The tissue sections were fixed in 10% neutral buffered formalin, embedded in paraffin, and cut into 4 μm thick sections. After deparaffinization and rehydration, the slices were stained with hematoxylin and eosin (H&E).

Rabbit anti-S-100 and a murine monoclonal antibody against HMB45 were purchased from DAKO (Carpinteria, CA, USA). Rabbit anti-microphthalmia-associated transcription factor (MITF) antibody was purchased from Bioss antibodies (cat. # bs-1990R; Woburn, MA, USA).

Immunohistochemical staining was performed according to a previously described procedure [15]. Briefly, antigen retrieval from deparaffinized sections was performed by autoclaving samples for 15 min in 10 mM citrate buffer (pH 6.0). Slides were then incubated for 30 min in 10% normal goat serum and subsequently incubated with antibodies overnight at 4°C. Immunohistochemical staining was performed using an ImmPRESS Polymerized Reporter Enzyme Staining System (Vector Laboratories Inc., Burlingame, CA, USA).

## Results

### CPI-613 induced autophagy in HS-MM cells

**Fig 1:**
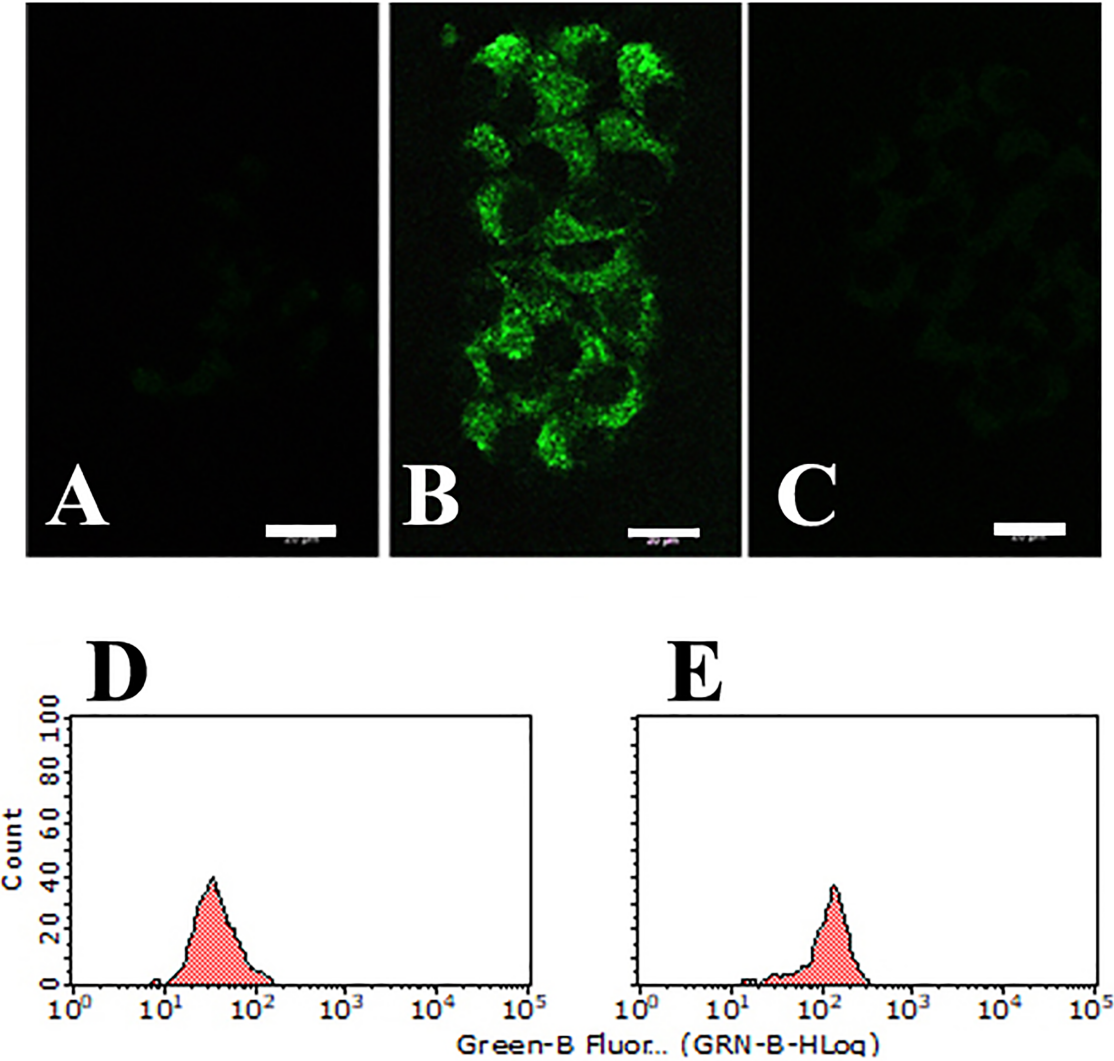
CPI-613 induced autolysosome formation in HS-MM CCS cells. DALGreen passes through the cell membrane of live cells and is incorporated into the autophagosome. After lysosome fuses with the autophagosome, enhanced incorporated DALGreen fluoresces as acidity increases, and this was visualized under a confocal fluorescence microscope. Lysosomal formation was not observed in HS-MM cells treated with vehicle only (A). Robust fluorescence intensity was observed in HS-MM cells treated with 1 μg/ml CPI-613 (B), but not in the presence of 10 μg/ml chloroquine (C) (Scale bar: 20 μm). Fluorescent intensity of HS-MM cells treated by vehicle alone (D) or CPI-613 and chloroquine (E) was analyzed with a Guava EasyCyte cell analyzer.

After a 16 h incubation with 1 μg/ml CPI-613, HS-MM cells exhibited morphological changes, which were highlighted by the formation of cytoplasmic vacuoles. As demonstrated in Fig 1, CPI-613 induced autolysosome in HS-MM cells.

### CPI-613 along with chloroquine induced cell death in HS-MM cells

**Fig 2:**
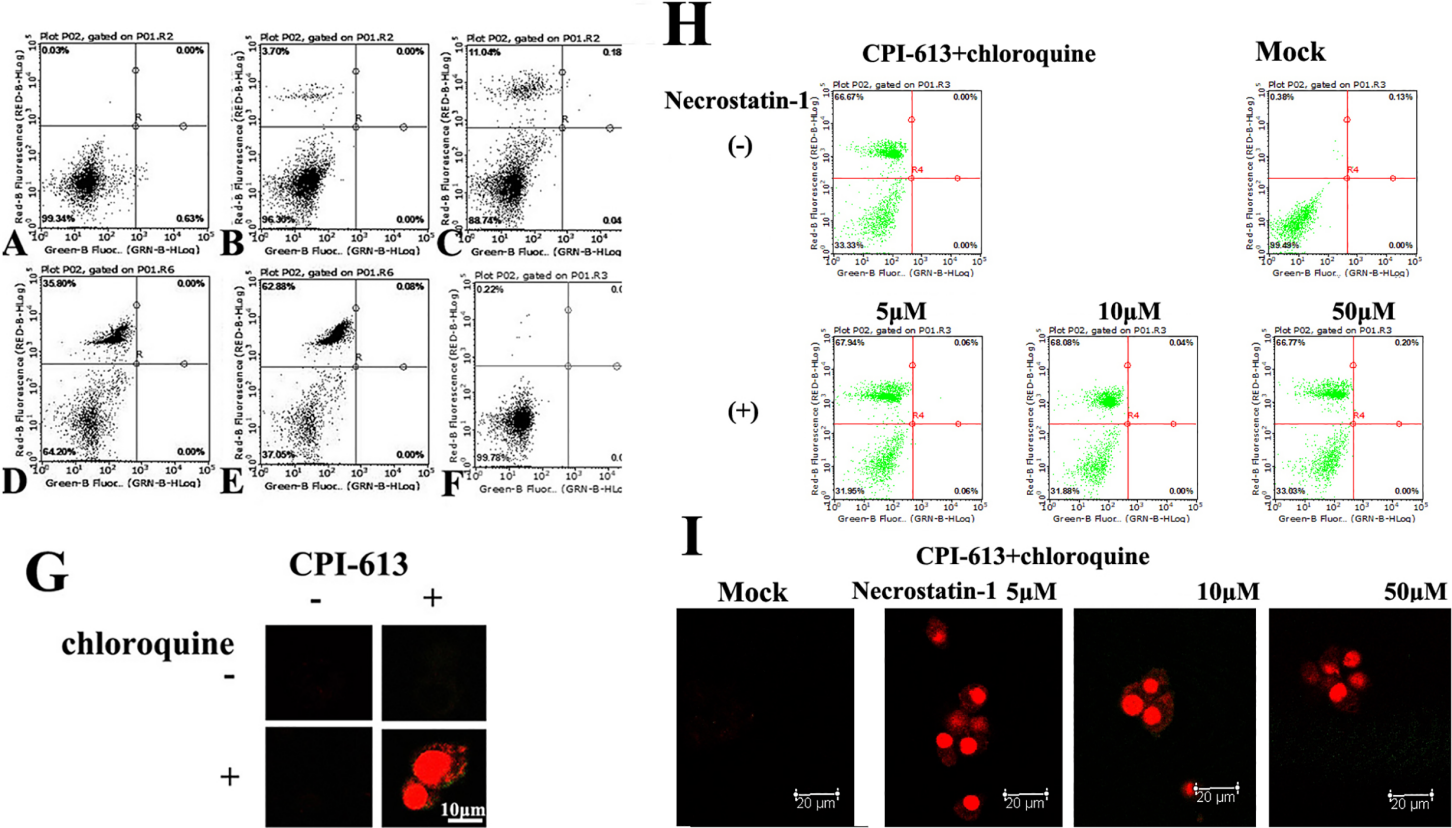
Double staining with fluorescence conjugated Annexin V and propidium iodide demonstrated that CPI-613 and chloroquine induced cell death in HS-MM CCS cells. A–F: Double staining was evaluated by a cell analyzer. No or little propidium iodide staining was found in control (A) or 10 μg/ml CPI-613 treated HS-MM cells for 4 h (B). Note the propidium iodide staining in C (100 ng/ml CPI-613), D (1 μg/ml CPI-613), and E (10 μg/ml CPI-613) were in the presence of 10 μg/ml chloroquine. No significant propidium iodide staining was found when cells were treated with 10 μg/ml chloroquine without CPI-613 treated cells (F). G: Double staining was visualized under a confocal fluorescence microscope. Note the robust nuclear staining with propidium iodide in the presence of 1 μg/ml CPI-613 and 10 μg/ml chloroquine (Scale bar: 10 μm). H: Double staining was evaluated by a cell analyzer. A necroptosis inhibitor, necrostatin-1 (5, 10, or 50 μM) did not affect death of HS-MM cells induced by a combination of 1 μg/ml CPI-613 and 10 μg/ml chloroquine. Mock mean data from untreated HS-MM cells stained by Annexin V and propidium iodide. I: Double staining was visualized under a confocal fluorescence microscope. Necrostatin-1 did not alter the cell death status induced by 1 μg/ml CPI-613 and 10 μg/ml chloroquine (Scale bar: 20 μm). Little Annexin V staining was observed, which indicates that CPI-613, along with chloroquine, induced necrosis in HS-MM CCS cells.

Cancer cells prevent the toxic buildup of cellular waste products by autophagy [15, 16]. Subsequently, we asked whether CPI-613 along with chloroquine, which blocks lysosome function and the degradation of autophagy cargo, impaired the growth of HS-MM cells. Surprisingly, CPI-613, along with chloroquine, induced cell death in HS-MM in a CPI-613 dose-dependent manner (Fig 2C, 2D and 2E). Since a necroptosis inhibitor, necrostatin-1 did not affect cell death, noted as Annexin V negative and PI positive (Fig 2H and 2I), we assume that CPI-613 along with chloroquine induced necrotic cell death.

### Orthotropic xenoplanted model of CCS in SCID-beige mice

**Fig 3:**
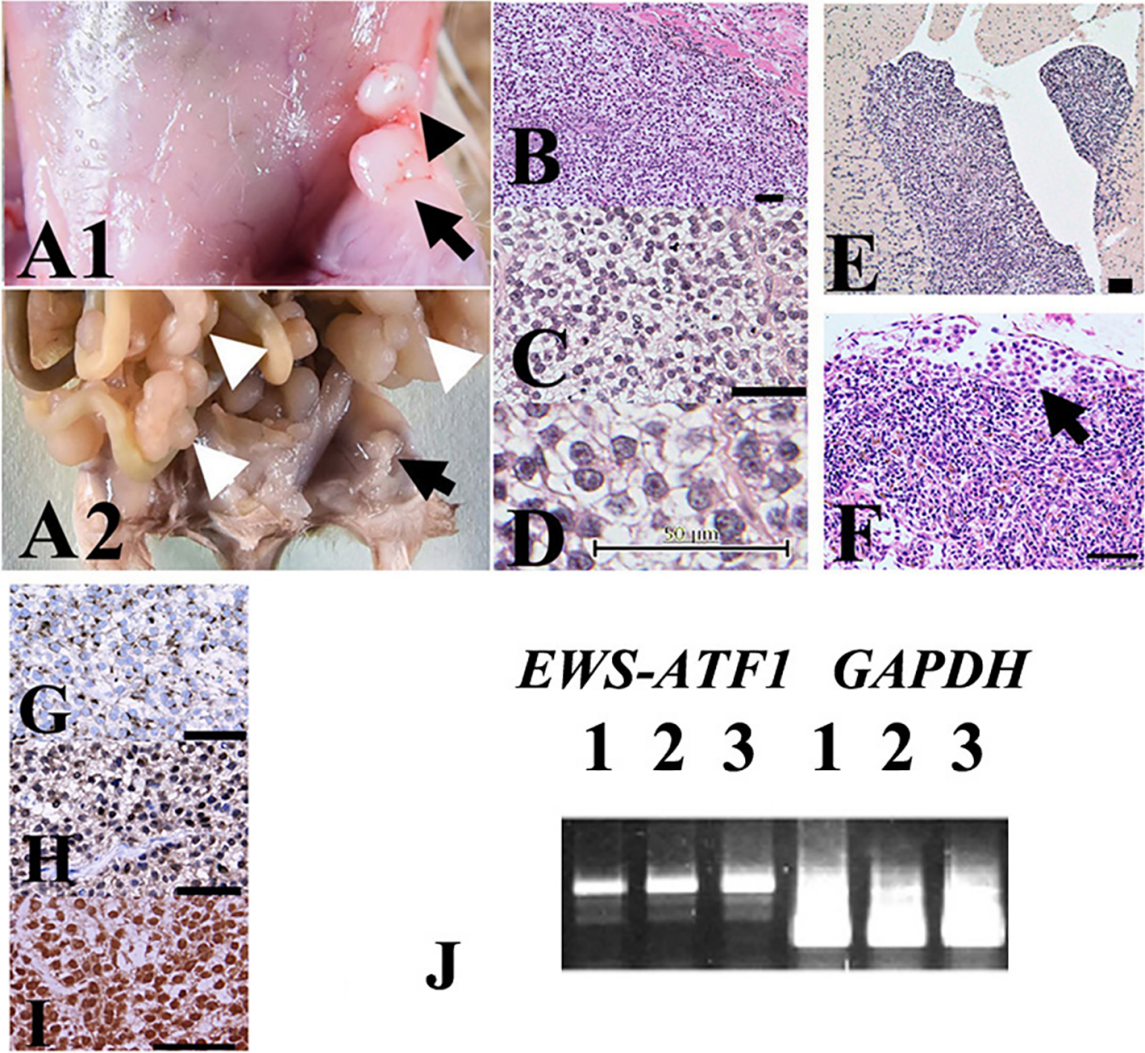
Growth of xenoplanted HS-MM cells with the *EWS-ATF1* fusion gene at the injection sites and metastatic lesions. Distant and lymphatic metastases were detected in all inoculated SCID-beige mice. Local lymphatic invasion is indicated by the black arrowhead near the primary injection site in the soft tissues of the thighs (black arrow in A1). Multiple disseminated tumors in the mesentery (white arrow heads in A2) were observed in addition to that in the subcutaneous injection site in the left thigh (black arrow). (B–D) HE staining of tissue sections from the injection site showed a nesting/sheet arrangement of tumor cells, with clear cytoplasm and prominent nucleoli. (D) Metastasis to the left ventricle of the heart (E) and the marginal sinus of the remnants of a lymph node (F) are shown. The arrow indicates the tumor cells in the marginal sinus of the lymph node (F). Immunohistochemical staining results showed that the tumor cells exhibited cytoplasmic staining with anti-HMB45 (G) and nuclear staining with anti-S-100 (H) and anti-MITF (I) antibodies, consistent with previous reports on CCS of soft tissues. Scale bars = 50 μm in B–I. (J) Representative RT-PCR results. Expression of the *EWSR1-ATF1* fusion transcript was observed in the primary injected site tumor (lane 1), ascites (lane 2), and distant metastasis to the lungs (lane 3) in SCID-beige mice. An original, uncropped, and unadjusted image of the agarose gel following electrophoresis is shown in S1 Fig.

Notably, lymph node metastasis and distant metastasis were detected at eight weeks in the liver, lungs, heart, and/or peritoneum of all inoculated SCID-beige mice. Peritoneal dissemination lesions were resected, minced, cultured, and transplanted into SCID-beige mice. After four repeated transplantations, accelerated cell growth was observed in the soft tissues of the thighs. Lymphatic metastasis and distant metastasis were detected within four weeks (Fig 3A). Both histopathological and molecular examination revealed that tumors exhibited a phenotype that was consistent with human CCS comprising polygonal cells containing abundant, clear cytoplasm bordered by thin fibrous septa, which are often observed in human CCS cases (Fig 3B–3D). Tumor cells showed positive staining with anti-S-100, -HMB-45, and -MITF antibodies, consistent with typical CCS observations (Fig 3G–3I).

We also examined the expression of the *EWSR1-ATF1* fusion gene in metastatic HS-MM cells. As demonstrated in Fig 3J, RT-PCR demonstrated *EWSR1-ATF1* fusion gene expression in both primary injected and metastatic HS-MM cells.

We concluded that the present orthotropic metastatic tumor model was useful in evaluating new and advanced therapies for CCS.

### Tumor suppressor effect of CPI-613 in conjunction with chloroquine in vivo

**Fig 4:**
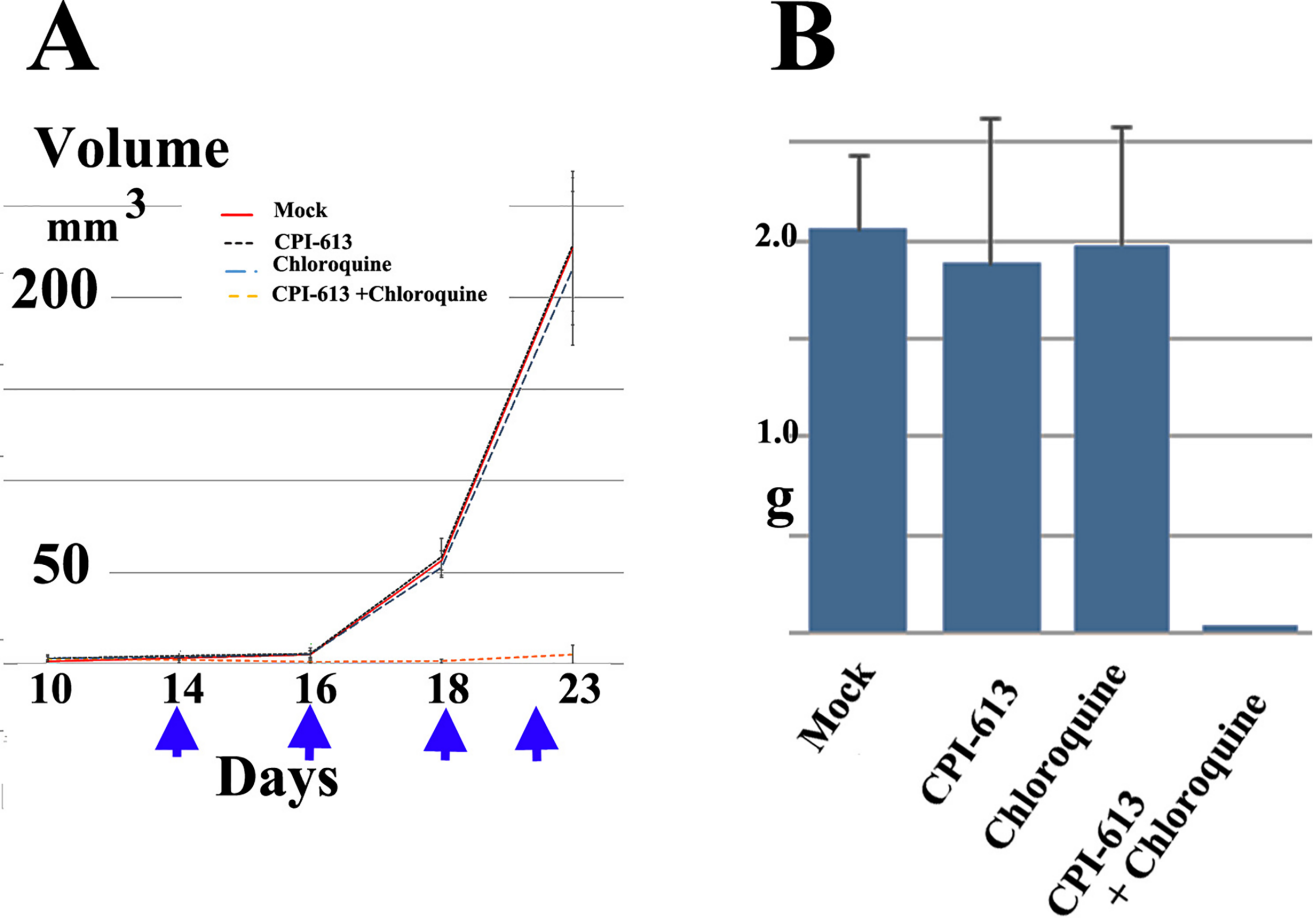
A combination of CPI-613 and chloroquine significantly suppressed tumor growth in an orthotropic metastatic tumor model of CCS. A: Tumor volume at injection site with intraperitoneal injection of vehicle only, (Mock), CPI-613, chloroquine, or a combination of CPI-613 and chloroquine. Intraperitoneal administration of CPI-613 (25 mg/kg) and chloroquine (50 mg/kg) significantly decreased tumor growth at the injection site compared to control (Student's *t*-test, *P* < 0.01 at days 18 and 23). Arrows indicate day of injection. Data are expressed as means ± SD (n = 5). B: Total weights of collected, disseminated mesenteric tumors after seven days from the last CPI-613 and chloroquine injections. Data are expressed as means ± SD (n = 5). Student’s *t*-tests were performed to determine statistically significant differences among groups. A combination of CPI-613 and chloroquine significantly reduced intraperitoneal metastasis compared to control (Student's *t*-test, *P* < 0.01).

We next attempted to determine whether a combination of CPI-613 and chloroquine had an inhibitory effect on tumor progression in the present murine model. Notably, intraperitoneal injection of CPI-613 and chloroquine not only decreased tumor growth at the primary injection site but also abrogated metastasis (Fig 4). No significant tumor suppressor function was found with CPI-613 or chloroquine alone (Fig 4). Notably, tumor suppression was observed in all five treated mice in this study, in both female (Fig 4) and male mice (S3 Fig).

## Discussion

CPI-613 is an analog of lipoic acid, which strongly disrupts mitochondrial metabolism through inhibiting the TCA cycle [6]. Despite the early dogma that cancer cells bypass the TCA cycle and solely use anaerobic glycolysis, recent advances demonstrate that certain cancer cells still utilize the TCA cycle for energy production and macromolecule synthesis [17]. Furthermore, CPI-613 disrupts the redox balance in cancer cell mitochondria [7].

In the present study, we first noted that CPI-613 markedly increased autolysosome formation (Fig 1B, 1C and 1F), but with a small amount of cell death (Fig 2B). Notably, chloroquine impaired autolysosome formation, or the fusion of autophagosomes with lysosomes and induced cell death in CPI-613-treated HS-MM CCS cells (Fig 2C–2E). These findings indicate that insufficient removal and degradation of CPI-613-induced autophagy cargo resulted in cell death in HS-MM CCS cells. Since Annexin V-positive apoptosis was not observed following CPI-613 and chloroquine treatment, the tumor cell death observed may be due to necrosis. In line with this idea, extensive studies to detect apoptosis (i.e., through apoptotic DNA fragmentation) have failed in CPI-613 and chloroquine-treated HS-MM cells (data not shown). It is generally accepted that cells with defects in apoptosis and autophagy fail to tolerate metabolic stress, undergo metabolic catastrophe, and die by necrosis [18, 19].

CPI-613 is well-tolerated by recent phase I clinical trials [8, 9]. In addition, chloroquine has long been used in the treatment or prevention of malaria, and has recently been applied to patients with autoimmune disorders, such as rheumatoid arthritis and lupus erythematosus [20]. Clearly, tolerance of both CPI-613 and chloroquine is beneficial for their clinical application. Therefore, we attempted to examine the effect of CPI-613 along with chloroquine on CCS progression *in vivo*. However, to the best of our knowledge, there are no animal models with high metastatic rates (i.e., animal models that mimic human CCS progression). Indeed, our own previous study demonstrated that BALB/c nude mice xenoplanted with cultured CCS cells showed no metastasis [11].

Next, we developed a murine model, the first animal model for CCS with a high rate of metastasis using SCID-beige mice. SCID-beige is a double-mutant mouse strain with impaired lymphoid development and weak NK (Natural Killer) cell activity [21]. Notably, SCID-beige mice allow for tumor cell growth and the metastasis of cell lines derived from various human cancers, including breast cancer [22], pancreatic cancer [23], and Ewing sarcoma [24]. Although further study is needed, we speculate that impaired NK cell function is linked to the metastatic microenvironment in SCID-beige mice.

As demonstrated in Fig 3, xenoplanted tumor cells exhibited the typical histopathological features of CCS and immunohistochemical profiles (i.e., positive for HMB45, S-100, and MITF). Moreover, tumor cells appeared to harbor the *EWS-ATF1* fusion gene, which is a canonical feature of CCS [25], at the primary injection site, ascites, and distant metastatic sites (Fig 3J). The similarity to the pathobiological phenotype and genetic background with similar progression pattern to human CCS may verify the present tumor model as an orthotropic metastatic CCS model.

Using a newly generated orthotropic metastatic model, we examined the efficacy of utilizing CPI-613 against CCS. As demonstrated in Fig 4, CPI-613 along with chloroquine significantly suppressed tumor growth and the metastasis of CCS cells in xeoplanted male SCID-Beige mice.

In conclusion, a combination of CPI-613 and chloroquine may have therapeutic potential for the treatment of patients with CCS, especially before or after surgical excision of the tumor(s) to control the local progression, recurrence, and metastasis.

## Supporting information

S1 Fig Supporting information for Fig 3. **Full scans of Fig 3J.** Original, uncropped, and unadjusted image of agarose gel following electrophoresis with λHINDIII DNA size marker.
(TIF)

S2 Fig Supporting information for Fig 1. **CPI-613 induced autolysosome formation in HS-MM CCS cells.** DALGreen passes through the cell membrane of live cells and is incorporated into the autophagosome. After a lysosome fuses with the autophagosome, enhanced incorporated DALGreen fluoresces as the acidity increases, and this was visualized under a confocal fluorescence microscope. Lysosomal formation was not found in HS-MM cells treated with vehicle only (A). Robust fluorescence intensity was found in HS-MM cells treated by 1 μg/ml CPI-613 (B and C), but not in the presence of 10 μg/ml chloroquine (D). Fluorescent intensity of HS-MM cells treated by vehicle alone (E) or CPI-613 and chloroquine (F) was analyzed with a Guava EasyCyte cell analyzer.
(TIF)

S3 Fig Supporting information for Fig 3. **A combination of CPI-613 and chloroquine significantly suppressed tumor growth in an orthotropic metastatic tumor model of CCS.** A: Intraperitoneal administration of CPI-613 (25 mg/kg) and chloroquine (50 mg/kg) significantly decreased tumor growth at the injection site and reduced the metastasis of HS-MM cells in SCID-beige male mice. Arrow indicates a day of injection of CPI-613 and chloroquine (two times weekly). **B:** Total weights of collected, disseminated mesenteric tumors after seven days from the last CPI-613 and chloroquine injections. Data are expressed as means ± SD (n = 5). Student’s *t*-tests were performed to determine statistically significant differences among groups (*P* < 0.01). **C:** Representative mice are shown. Note the reduction in metastasis of CPI-613 and chloroquine treated HS-MM cells (indicated as CPI-613) compared to those of control mouse. White arrow indicates the distant metastasis.
(TIF)

S4 Fig Supporting information for Fig 3. **Expression of the EWSR1-ATF1 fusion transcript.** Expression of the *EWSR1-ATF1* fusion transcript was observed in distant metastasis to the lung (indicated as lung), ascites, and the primary injected site tumor of all five HS-MM transplanted SCID-beige mice (numbered as 1, 2, 3, 4, and 5). Image of agarose gel following electrophoresis with λHINDIII DNA size marker.
(TIF)

S1 Table Supporting information for Fig 4A. Longest diameter, shortest diameter, and calculated tumor volumes.
(XLSX)

S2 Table Supporting information for Fig 4B. Total weights of collected, disseminated mesenteric tumor.
(XLSX)

S3 Table Supporting information for S3A Fig. Tumor volumes of control and CPI613-Chloroquine treated mice.
(XLSX)

S4 Table Supporting information for S3B Fig. Total weights of collected, disseminated mesenteric tumor.
(XLSX)
