# Supplementary figures and images for: Therapeutic potential of CPI-613 for targeting tumorous mitochondrial energy metabolism and inhibiting autophagy in clear cell sarcoma
Source: PLoS One. 2018 Jun 7;13(6):e0198940. doi: 10.1371/journal.pone.0198940 (PMC5991736; doi:10.1371/journal.pone.0198940)

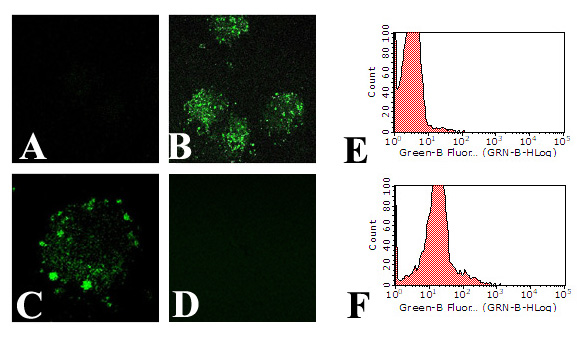

Supplement: S2 Fig — CPI-613 induced autolysosome formation in HS-MM CCS cells. DALGreen passes through the cell membrane of live cells and is incorporated into the autophagosome. After a lysosome fuses with the autophagosome, enhanced incorporated DALGreen fluoresces as the acidity increases, and this was visualized under a confocal fluorescence microscope. Lysosomal formation was not found in HS-MM cells treated with vehicle only (A). Robust fluorescence intensity was found in HS-MM cells treated by 1 μg/ml CPI-613 (B and C), but not in the presence of 10 μg/ml chloroquine (D). Fluorescent intensity of HS-MM cells treated by vehicle alone (E) or CPI-613 and chloroquine (F) was analyzed with a Guava EasyCyte cell analyzer. (TIF) [file pone.0198940.s002.tif]

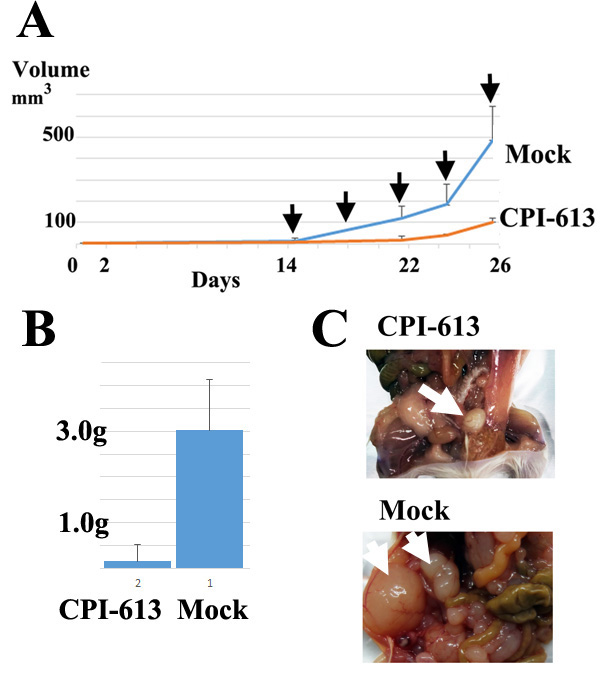

Supplement: S3 Fig — A combination of CPI-613 and chloroquine significantly suppressed tumor growth in an orthotropic metastatic tumor model of CCS. A: Intraperitoneal administration of CPI-613 (25 mg/kg) and chloroquine (50 mg/kg) significantly decreased tumor growth at the injection site and reduced the metastasis of HS-MM cells in SCID-beige male mice. Arrow indicates a day of injection of CPI-613 and chloroquine (two times weekly). B: Total weights of collected, disseminated mesenteric tumors after seven days from the last CPI-613 and chloroquine injections. Data are expressed as means ± SD (n = 5). Student’s t-tests were performed to determine statistically significant differences among groups (P < 0.01). C: Representative mice are shown. Note the reduction in metastasis of CPI-613 and chloroquine treated HS-MM cells (indicated as CPI-613) compared to those of control mouse. White arrow indicates the distant metastasis. (TIF) [file pone.0198940.s003.tif]

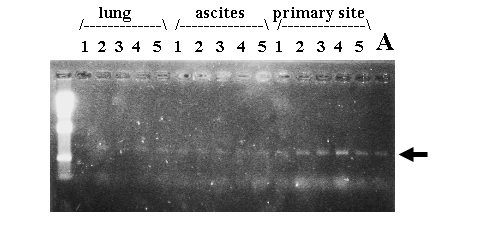

Supplement: S4 Fig — Expression of the EWSR1-ATF1 fusion transcript. Expression of the EWSR1-ATF1 fusion transcript was observed in distant metastasis to the lung (indicated as lung), ascites, and the primary injected site tumor of all five HS-MM transplanted SCID-beige mice (numbered as 1, 2, 3, 4, and 5). Image of agarose gel following electrophoresis with λHINDIII DNA size marker. (TIF) [file pone.0198940.s004.tif]
